# Primary care and pattern of skin diseases in a mediterranean island

**DOI:** 10.1186/1471-2296-7-6

**Published:** 2006-01-31

**Authors:** Emmanouil K Symvoulakis, Konstantin Krasagakis, Ioannis D Komninos, Ioannis Kastrinakis, Ioannis Lyronis, Anastasios Philalithis, Androniki D Tosca

**Affiliations:** 1 Department of Social Medicine, Faculty of Medicine, University of Crete, Greece; 2 Department of Dermatology, University General Hospital of Heraklion, Crete, Greece

## Abstract

**Background:**

In Greece where primary health care services are not fully developed, patients with simple or minor conditions have to attend to hospitals to be treated. We analysed the data of patients with cutaneous disorders attending the tertiary referral hospital on the Island of Crete, with the aim to identify the most common conditions that patients complain of, in order to define the areas where the education of General Practitioners in Dermatology must focus.

**Methods:**

All patients attending the Dermatology ambulatory office in the Emergency Department of the University General Hospital of Heraklion from January 2003 to December 2003 were included in this retrospective analysis. The medical records of the patients (history, physical examination and laboratory investigations) were analysed to ascertain the diagnosis and the management of cases. All patients were evaluated by qualified dermatologists.

**Results:**

A total of 3715 patients attended the Dermatology Clinic. Most patients were young adults in the age group 21–40 years (38.4%), and the male to female ratio was 1 to 1.2. Allergic skin diseases, mostly dermatitis and urticaria (35.7%) were the most common for attendance, followed by infectious diseases (26.1%) and insect bites (10.2%). Inflammatory and autoimmune disorders accounted for 7.9% of the cases. Pruritus of unknown origin was diagnosed in 6.3% of patients. Skin tumors were detected in 2.7%. The management of the vast majority of cases (85.0%) consisted of advice with or without a prescription, while only 4.8% of patients required admission.

**Conclusion:**

Allergic and infectious skin diseases were the most common cutaneous diseases in patients attending this tertiary University hospital, while the management of most patients did not require specialised care. On the basis of the present data, the training of primary health care providers in Dermatology should emphasize these common conditions, with the aim of improving primary care and alleviating the burden on hospital care.

## Background

During the last three decades, dramatic changes have occurred in health care provision in western countries. Managed care plans attempt to reduce costs by encouraging primary health care providers to handle a greater and wider range of conditions [1]. In the U.S.A., approximately 6% of outpatient visits are for dermatological diseases and non-dermatologists treat a high percentage of these patients [2,3]. In Greece, primary health services are still not fully developed, in particular in cities, where there is a lack of General Practitioners. In Crete, there are very few Dermatologists working as private practitioners in order to provide care to patients who would refer, self-pay, themselves directly to a specialist. Therefore, most patients with any type of skin disorder attend the Dermatology ambulatory office in the Emergency Department of the University General Hospital in order to be diagnosed and treated. The aim of this study is to determine the type (diagnosis and classification) of skin disorders that patients present with and to ascertain how they are managed. It is hoped that this approach may help to improve the education of primary health care providers [4] by focusing on diagnosis and treatment of the most common of cutaneous diseases.

## Methods

All patients seen at the Dermatology ambulatory office in the Emergency Department of the University General Hospital of Heraklion from January 2003 to December 2003 were included in this retrospective analysis. From the medical records, history, physical examination and whenever necessary, laboratory investigations were analysed. All patients were evaluated by qualified dermatologists. Sex, age, clinical diagnoses and seasonal fluctuations of the most common skin problems were recorded. The management of the patients was also studied. Descriptive statistics were carried out.

## Results

**Figure 1:**
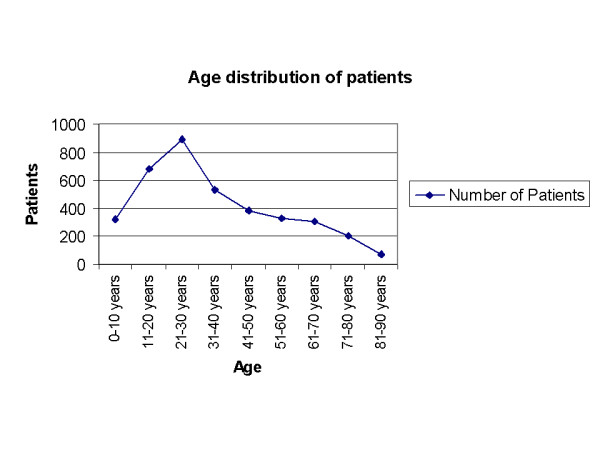
Age distribution of all patients attended the Dermatology ambulatory office in a year.

**Figure 2:**
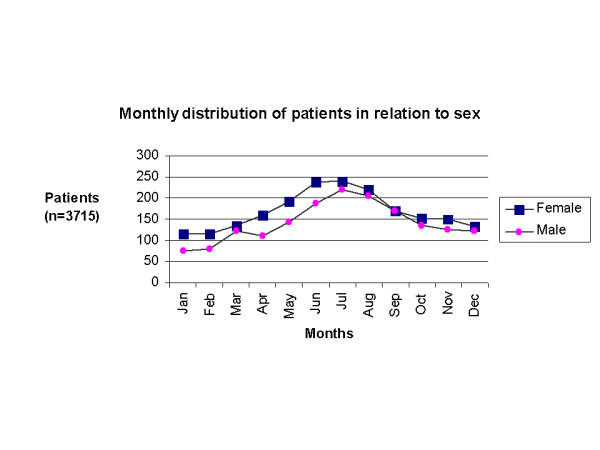
Monthly distribution of all patients attended the Dermatology ambulatory office in relation to sex.

A total of three thousand seven hundred fifteen patients (3715) attended the Dermatology department. Thirty-eight per cent of patients were in the age group 21–40 years, and the male to female ratio was 1:1.2. Age distribution of cases is shown in Figure 1. The monthly distribution of patients analyzed by sex is shown in Figure 2. The pattern and the relative frequency of skin diseases are shown in Tables 1, 2. More than one third of the patients (35.7%) attended the hospital with a cutaneous manifestation of an allergic disorder [Table 1]. The most common were dermatitis and eczematous disorders that accounted for 18.1% (674 patients), followed by acute urticaria or exacerbations of chronic urticaria (14.1%, 524 patients) and drug eruptions (85 patients, 2.3%), whereas erythema nodosum and erythema multiforme accounted only for 0.6% each (21 and 23 patients respectively). A breakdown of dermatitis subtypes showed that contact dermatitis is the most common (32.2%, 217 of dermatitis cases), followed by atopic dermatitis (20.1%, 135 of cases). Dyshidrotic and seborrheic dermatitis were observed in 10.3% (69 cases) and 7.4% (50 cases) respectively. Unclassified dermatitis was seen in 30.0% (202 patients). Infectious diseases with cutaneous manifestations were observed in 26.1% of the patients. Bacterial infectious diseases accounted for 9.1% (337 patients), followed by viral exanthemas (7.3%, 271 patients) and fungal infections (5.5%, 203 patients), whereas viral warts, parasitic or venereal diseases accounted for less than 2% of the examined cases [Table 1].

**Table 1:** Distribution of cases in relation to the type of skin disorder.

**Type of skin disorder**	**Cases (n)**	**Percentage (%)**
**Allergic skin disorders**	**1327**	**35.7**
Dermatitis	674	18.1
Urticaria	524	14.1
Drug eruptions	85	2.3
Erythema multiforme	23	0.6
Erythema nodosum	21	0.6
**Infectious skin disorders**	**968**	**26.1**
Bacterial infections	337	9.1
Viral exanthemas	271	7.3
Fungal infections	203	5.5
Viral warts	59	1.6
Parasitic diseases	52	1.4
Venereal diseases	46	1.2
**Inflammatory and autoimmune skin disorders**	**294**	**7.9**
Acne	85	2.3
Pityriasis rosea	67	1.8
Connective tissue diseases	52	1.4
Psoriasis	46	1.2
Lichen Planus	22	0.6
Bullous diseases	22	0.6
**Miscellaneous group**	**1126**	**30.3**
Insect bites	379	10.2
Pruritus of unknown origin	234	6.3
Burns	63	1.7
Skin tumors	100	2.7
Aktinic keratosis	46	1.2
Ulcers	26	0.7
Pigmentary disorders	33	0.9
Alopecia	22	0.6
Others	53	1.4
No skin findings	96	2.6
No clinical diagnosis	74	2.0
**Total**	**3715**	**100**

**Table 2:** Seasonal relative frequency of the most common skin disorders.

**Common skin disorders**	**Seasons**			
	
	**Spring (%)**	**Summer (%)**	**Autumn (%)**	**Winter (%)**
**Dermatitis**	19.8	16.8	17.8	19.1
**Urticaria**	16.7	12.6	12.8	15.7
**Insect Bites**	8.0	14.9	9.4	4.7
**Bacterial Infections**	8.0	10.8	8.4	7.9
**Viral Exanthemas**	7.2	5.2	9.6	8.6
**Fungal Infections**	4.1	6.4	6.3	4.4
Pruritus*	5.0	4.3	9.3	7.8
**Others**	31.2	29.0	26.4	31.8
**Total**	100	100	100	100

* Of unknown origin

Exacerbations of mostly chronic autoimmune and inflammatory skin disorders were the reason of the consultation in 7.9% of the patients [Table 1]. Acne accounted for 2.3% of them, psoriasis for 1.2%, connective tissue disorders for 1.4%, lichen planus for 0.6% and bullous diseases for 0.6%. Pityriasis rosea, an acute onset disease of unknown origin, was diagnosed in 1.8% of the patients. Several other causes accounted for 30.3% of outpatient visits at the emergency department [Table 1]. Insect bites – especially in summer months – were diagnosed in 10.2% of the patients, whereas pruritus of unknown origin in 6.3%. Skin tumors were seen in 2.7% of the patients and actinic keratoses in 1.2%. Burns were observed in 1.7%, ulcers (mostly located at the legs) in 0.7%, whereas pigmentary disorders and alopecia in 0.9% and 0.6% respectively. Clinical diagnosis could not be established in 2.0% of the patients and 2.6% had no skin findings or symptoms. The seasonal distribution of seven most common skin diseases accounting for approximately 70% of all diagnosed patients is seen in Table 2.

The tabulation of the work out of the cases revealed that 3156 patients (85.0%) were received hygienic and dietary advice with or without a prescription for drugs (Table 3). A re-evaluation plan as outpatients was planned in 246 patients (6.6%). One hundred and seventy eight patients (4.8%) were admitted to the clinic for further diagnostic procedures and treatment. For 39 cases (1.0 %) referral to an internist for further was necessary.

**Table 3:** Management of all patients who attended the Dermatology ambulatory office.

**Type of management**	**Patients**	
	
	**n = 3715**	**(%)**
**Medical advice with or without prescription for drugs**	3156	85.0
**Outpatient plan consultation**	246	6.6
**Hospitalisation**	178	4.8
**Internal medicine specialist's consultation**	39	1.0
**None (absence of skin finding)**	96	2.6

## Discussion

The Department of Dermatology in the University General Hospital of Heraklion is the only tertiary referral department in the island of Crete (population 600 000). In addition, there is a lack of a full-functioning primary health care provision, especially in the urban areas of the island and the number of dermatologists providing first contact care is limited. First manifestation or acute exacerbation of a chronic existing skin disease is therefore a common reason for patients to seek care at the University Hospital of Heraklion. This study was therefore conducted to describe the pattern of those dermatological diseases that could be managed by primary care physicians if they had received appropriate training.

The highest number of cases observed was dermatitis followed by acute urticaria, which are both skin diseases based on a background of immediate or delayed hypersensitivity. This observation correlates well with other studies that report allergic skin diseases as the most common cause to seek care from a health provider [4-6]. The highest rate of allergic skin disorders was observed during Spring. Among the allergic skin diseases observed, dermatitis was predominant. The breakdown of dermatitis subtypes has revealed a high frequency of contact dermatitis. Shenefelt reports contact dermatitis as the first among the types of dermatitis, seen in a similar percentage of 30% [7]. This was followed by atopic dermatitis while dyshidrotic dermatitis was found third, in contrast with other studies that report a low frequency of dyshidrotic dermatitis [5]. Urticaria represents a disease with a wide spectrum of causes. The patients attending our hospital suffered from either acute urticaria or exacerbations of chronic urticaria. Patients with a clear cause for their urticaria, who were mostly acute urticaria patients, were advised to avoid the causative agent, i.e. the suspected food or drug. Patients with chronic urticaria, however, after treatment of the relapse episode were referred to a specialist for clarification of the complex aetiology of the disease and for further follow up. Drug eruptions, erythema nodosum, erythema multiforme and Stevens Johnson syndrome represented only a small percentage of patients seeking for care. Several of these cases represented undiagnosed or insufficiently treated patients with more severe course that necessitated hospitalisation for identification of the causative agent and further treatment. However the total hospitalisation rate of all cases was only 4.8%.

Infectious skin disorders were the second most common disease group in this study. Bacterial skin infections ranked higher among cutaneous infections. The highest rate of bacterial skin infections was observed during Summer, probably due to heat and humidity. Viral exanthemas were the second most common reason for patients with infections seeking care, followed by fungal diseases. Viral exanthemas showed the maximum frequency during Autumn and Winter, while fungal skin infections presented their highest rate during Summer and Autumn. Parasitic diseases were observed in a very low frequency and this might be due to misdiagnosis and /or an overlap with pruritus of unknown origin that was detected in a relatively high percentage. Infectious and parasitic diseases are less common in relation to the findings of other studies in Africa where the socio-economic conditions are different [5,6]. Insect bites are the third reason in total for patients seeking dermatological care (10.2% of the total cases) and the maximum frequency was observed during Summer. During Winter the frequency of patients with insect bites decreased to 4.7%.

Although skin tumors were diagnosed in a percentage of 2.7%, this finding cannot be confirmed without a biopsy. For the same reason, the relative frequency of benign and malignant lesions cannot be accurately determined despite our initial clinical hypothesis. Julian reports an incidence of skin cancer and actinic keratoses detected in 3% and 3.3% respectively [8]. In the current study, actinic keratoses were observed in 1.2% of patients (46). On the other hand, we believe, from our experience, that the majority of patients with a suspicion of a skin tumor are seen in the regular Dermatology outpatient settings since the onset of their symptoms or signs is progressive and chronic.

Inflammatory and autoimmune disorders represent mostly diseases with a chronic and relapsing course. Psoriasis was the leading cause of dermatological consultation for papulosquamous diseases, seen in 1.2% of the total cases examined (46 cases). Julian reports psoriasis in a percentage of 2.6% and other studies suggest psoriasis as the chronic dermatological disorder that affects 1 to 2% of the population [8,9]. Acne is a common skin disorder that affects susceptible pilosebaceous follicles of mainly teenagers and young adults [10,11]. Acne was seen in a percentage of 2.3% of all cases in our study. The majority of acne patients had already attended a prior consultation. Acne is found worldwide and is more severe in males, with clinical evidence indicating a familial trait [12]. Psychologic and emotional stress may accompany this skin condition [13]. In 2,6% of cases no skin finding was detected during the clinical examination. A transient minimal skin lesion may explain this.

A breakdown of care management showed that 85.0% of patients required medical advice with or without prescription of drugs while only in 12.4% was hospital management necessary (Table 3). Although some of the first group might still require referral to a specialist, we believe that our results support the hypothesis that first contact care could be provided by a General Practitioner or other primary health care provider.

## Conclusion

Primary care physicians should have the working knowledge to handle the most common skin diseases in order to facilitate the management of common dermatological problems and to recognise those cases that require further referral. This may decrease the rate of hospital visits and reduce costs. Studies similar to ours will help confirm the most common conditions seen in dermatology and will provide the guidelines for the type of skin disorders that should be incorporated into the training program of General Practitioners [4]. Despite the fact that skin disease is often associated with less expensive diagnostic and therapeutic procedures and limited mortality, skin disorders are a leading cause of disability in the society [14]. The pattern of skin diseases is, among other parameters, an index of community development and of quality of the provided care. An effort to improve primary care and alleviate the burden on hospital care should be the target of a health policy.

## Competing interests

The author(s) declare that they have no competing interests.

## Authors' contributions

EKS, AP, KK and ADT were involved with the study conception. EKS, IDK, IK and IL performed the data acquisition and interpretation. KK and EKS were involved in revising the article for important intellectual content. All authors read and approved the final manuscript.

## Pre-publication history

The pre-publication history for this paper can be accessed here:


